# Incipient Tuberculosis

**Published:** 1907-06

**Authors:** 


### INCIPIENT TUBERCULOSIS.

Hay deals with the question of the early diagnosis of consumption, especially as met with in despensaries. Cough is almost inviriably present as one of the first signs; it is due in some cases to congestion or catarrh of the lung, to pleurisy, or to early involvement of the larnyx. The cough is usually short and ineffectual. Hæmoptysis, if genuine, is of paramount importance in the history. It may occur before there are any demonstrable physical signs in the lungs, and when associated with a history of cough, is almost pathognomonic. It is rarely profuse in the early stages, sometimes being only streaks in the expectoration. It may be due to hyperæmia of the parenchyma or bronchial mucous membrane, to softening of a small focus of disease and the opening of a minute vessel, to ulceration of a tuberculous patch in the bronchial mucous membrane, etc. Pain and tenderness in the chest may be of no moment or may indicate pleurisy. Tachycardia without pyrexia, but with constitutional disturbance, is occasionally the first symptom, or sometimes the case is taken for one of chlorosis. In considerating the value of physical signs in the diagnosis, it must be borne in mind that (1) the localization of physical signs is of much greater moment than their quality; (2) it is a safe plan to attach much weight to the presence of certain signs, little weight to their absence; (3) it is always unwise to base a diagnosis on any one physical sign; and that (4) one very often meets with very definite variations from the normal in perfectly sound chests. Incipient pulmonary tuberculosis and influenza are frequently confused. The initial symptoms of tuberculosis are often diagnosed as influenza; an attack of influenza, with its consequent debility, may be followed by tuberculosis infection; and, finally, influenza may light up a quiescent tubercular lesion.—Lancet, April 12th, 1907.

